# Short-term biochemical outcomes after microwave ablation versus parathyroidectomy for primary hyperparathyroidism: an IPTW analysis

**DOI:** 10.3389/fendo.2026.1921824

**Published:** 2026-09-16

**Authors:** Le Yin, Feng Luo, Yalin Chai, Lijie Sun, Congjuan Luo

**Affiliations:** The Affiliated Hospital of Qingdao University, Qingdao, China

**Keywords:** inverse probability of treatment weighting, microwave ablation, parathyroid hormone, parathyroidectomy, primary hyperparathyroidism

## Abstract

**Objective:**

To compare the short-term biochemical efficacy and safety of microwave ablation (MWA) and parathyroidectomy (PTX) in patients with primary hyperparathyroidism (PHPT), and to identify independent predictors of treatment failure.

**Materials and methods:**

This retrospective cohort study included 173 consecutive patients with PHPT treated with MWA (n = 46) or PTX (n = 127) between January 2019 and January 2025. Covariate-balancing propensity score–derived inverse probability of treatment weighting (CBPS-IPTW) was used to balance baseline characteristics. Longitudinal changes in parathyroid hormone (PTH), calcium, and phosphorus were assessed using IPTW-weighted linear mixed-effects models, whereas biochemical treatment failure and safety outcomes were compared using weighted logistic regression with robust standard errors. IPTW-weighted Kaplan–Meier and Cox analyses were exploratory, and factors associated with treatment failure were evaluated using Firth penalized logistic regression.

**Results:**

After weighting, all covariates were well balanced, with absolute standardized mean differences <0.01. Both treatments produced substantial biochemical improvement. A significant treatment-by-time interaction was observed for PTH (P < 0.001), whereas calcium and phosphorus trajectories did not differ significantly. The weighted risks of treatment failure were 23.8% after PTX and 35.9% after MWA [odds ratio (OR), 1.80; 95% confidence interval (CI), 0.74–4.39], with an absolute risk difference of 12.1 percentage points (95% CI, −7.3 to 31.6). Exploratory Cox analysis showed no significant difference in documented treatment failure (hazard ratio, 1.73; 95% CI, 0.82–3.64). Weighted hypocalcemia risks were 9.2% and 15.5%, respectively (OR, 1.81; 95% CI, 0.50–6.56). Each doubling of baseline PTH was associated with approximately twofold higher odds of treatment failure (OR, 2.02; 95% CI, 1.39–4.09).

**Conclusion:**

Both MWA and PTX produced substantial short-term biochemical improvement. No significant difference in 6-month treatment failure was detected after CBPS-IPTW adjustment; however, wide confidence intervals precluded conclusions of equivalence or non-inferiority. Baseline PTH may help identify patients at increased risk of incomplete biochemical response, and MWA may be considered for carefully selected patients.

## 1 Introduction

Primary hyperparathyroidism (PHPT) is the third most common endocrine disorder and is characterized by hypercalcemia accompanied by elevated or inappropriately normal serum parathyroid hormone (PTH) levels despite hypercalcemia (1, 2). Sporadic PHPT is predominantly caused by a single benign parathyroid adenoma (approximately 85%), whereas multiglandular disease, including parathyroid hyperplasia or multiple adenomas, accounts for approximately 15% of cases. Parathyroid carcinoma is a rare cause, representing less than 1% of cases (3, 4). The excessive secretion of PTH in patients with PHPT results in a spectrum of systemic manifestations involving the skeletal, renal, cardiovascular, neuropsychiatric, and neuromuscular systems (5, 6). Between 2009 and 2020, the age- and sex-adjusted incidence of PHPT increased from 2.81 (95% confidence interval [CI], 2.55–3.09) to 4.28 (95% CI, 3.98–4.59) per 10,000 person-years, with the greatest increase observed among women aged 70 years or older (3, 7). Consequently, treatment is recommended for patients with symptomatic PHPT (8). In addition, asymptomatic patients remain at risk of disease progression; therefore, therapeutic intervention should also be considered for those with skeletal and/or renal involvement (4). Current international guidelines recommend parathyroidectomy (PTX) as the standard treatment for PHPT (4, 9, 10). Surgical removal of the hyperfunctioning parathyroid gland eliminates the source of excessive PTH secretion and achieves biochemical cure in the majority of patients (11, 12). However, a subset of patients is either unsuitable for surgery because of medical contraindications or unwilling to undergo an operative procedure. In recent years, thermal ablation techniques, including microwave ablation (MWA) and radiofrequency ablation (RFA), have demonstrated favorable efficacy in the destruction of hyperfunctioning parathyroid lesions and normalization of serum PTH and calcium levels (13). Nevertheless, although thermal ablation has shown considerable promise as a minimally invasive treatment for PHPT, the current evidence remains limited by relatively small sample sizes and short follow-up durations, and high-quality comparative data are still lacking. Therefore, the present study was conducted to systematically compare the efficacy and safety of microwave ablation and parathyroidectomy in patients with primary hyperparathyroidism and to identify independent risk factors associated with treatment failure, thereby providing evidence to inform individualized treatment selection and postoperative risk stratification.

## 2 Materials and methods

### 2.1 Study design and patients

This retrospective cohort study was conducted by reviewing the electronic medical records of patients with PHPT who underwent MWA or PTX at the Affiliated Hospital of Qingdao University between January 2019 and January 2025. Clinical, laboratory, imaging, procedural, pathological, and follow-up data were obtained from the hospital electronic medical record system. Only patients with sporadic PHPT and complete clinical data were included in the analysis. The biochemical diagnosis and localization of PHPT were established according to the recommendations of the International Workshop on the Management of Primary Hyperparathyroidism. Ultrasound-guided core-needle biopsy (CNB) was performed for all parathyroid lesions to obtain histopathological confirmation and exclude parathyroid carcinoma. Localization of hyperfunctioning parathyroid lesions was achieved using cervical ultrasonography and technetium-99m sestamibi (99mTc-sestamibi, MIBI) scintigraphy. In addition, all patients scheduled to undergo MWA underwent preprocedural laryngoscopic examination to exclude recurrent laryngeal nerve (RLN) dysfunction. Patient eligibility was determined according to the diagnostic criteria and management recommendations of the Fifth International Workshop on the Management of Primary Hyperparathyroidism. Patients with PHPT were considered eligible for intervention if they met at least one of the recommended criteria, including age <50 years; serum calcium concentration >1.0 mg/dL (0.25 mmol/L) above the upper limit of the normal reference range; skeletal involvement defined as a T-score ≤−2.5 at the lumbar spine, femoral neck, total hip, or distal one-third radius, or the presence of vertebral fractures; renal involvement including an estimated glomerular filtration rate (eGFR) <60 mL/min/1.73 m², nephrolithiasis, nephrocalcinosis, or increased risk of kidney stone formation; or hypercalciuria defined as 24-hour urinary calcium excretion >250 mg/day in women or >300 mg/day in men (4). For patients undergoing ultrasound-guided microwave ablation (MWA), additional selection criteria were applied: 1) the presence of at least one enlarged and clearly localized parathyroid lesion identified by preoperative imaging, including cervical ultrasonography and/or 99mTc-sestamibi scintigraphy; 2) a lesion with a safe and feasible ultrasound-guided puncture pathway for complete ablation; 3) absence of imaging or clinical evidence suggestive of parathyroid carcinoma; and 4) no definite evidence of extensive multiglandular disease requiring surgical exploration. The exclusion criteria were as follows: 1) secondary or tertiary hyperparathyroidism; 2) severe hypertension, heart failure, or severe coagulation disorders precluding intervention; and 3) suspected parathyroid carcinoma based on imaging findings or laboratory evaluation. A total of 173 patients met the eligibility criteria and were included in the final analysis. The study protocol was approved by the Institutional Ethics Committee of the Affiliated Hospital of Qingdao University (Approval No. QYFY WZLL 50197). Written informed consent for treatment was obtained from all patients. The requirement for additional informed consent for participation in this retrospective study was waived because no identifiable personal information was disclosed.

### 2.2 Data collection and variable definitions

The following variables were collected: age, sex, serum PTH, albumin-adjusted serum calcium, serum phosphorus, renal function, nephrolithiasis, nephrocalcinosis, maximum gland diameter, number of localized parathyroid lesions, treatment date, and treatment modality. Serum intact parathyroid hormone (PTH), total calcium, phosphorus, albumin, and creatinine concentrations were measured in the central laboratory of the Affiliated Hospital of Qingdao University using routine automated laboratory assays. PTH was reported in pg/mL, calcium and phosphorus in mmol/L, albumin in g/L, and creatinine in μmol/L. All measurements were interpreted according to the assay-specific reference intervals in use at the central laboratory during the study period. Renal function was assessed using the estimated glomerular filtration rate (eGFR), calculated with the 2021 Chronic Kidney Disease Epidemiology Collaboration creatinine equation without a race coefficient. Renal insufficiency was defined as an eGFR <60 mL/min/1.73 m². Nephrolithiasis and nephrocalcinosis were identified from preprocedural ultrasonography, computed tomography, or other documented renal imaging. Maximum gland diameter was defined as the largest sonographically measured diameter of the dominant parathyroid lesion. The number of localized glands was defined as the number of suspected hyperfunctioning parathyroid lesions identified during the preprocedural imaging assessment. All available repeated measurements obtained during the first 6 months after treatment were included in the longitudinal analysis.

### 2.3 Ultrasound-guided microwave ablation procedure

MWA was performed using a GE LOGIQ E9 or LOGIQ E20 ultrasound system (GE Healthcare, Milwaukee, WI, USA) equipped with ML6–15 or 9L linear-array transducers (frequency range, 4–10 MHz). Before ablation, contrast-enhanced ultrasound (CEUS) was performed in real time to evaluate the perfusion characteristics and extent of the hyperfunctioning parathyroid lesion. Patients were placed in the supine position with the neck extended by a cushion positioned beneath the shoulders to ensure adequate exposure of the anterior cervical region. After routine skin sterilization, local infiltration anesthesia was achieved using 1% lidocaine. Under real-time ultrasound guidance, while avoiding visible vascular structures identified by color Doppler imaging, a sterile syringe was advanced into the extracapsular space adjacent to the thyroid gland. Approximately 20 mL of normal saline was injected to establish a hydrodissection barrier measuring greater than 5 mm between the parathyroid lesion and adjacent critical structures, including the recurrent laryngeal nerve, esophagus, and major cervical vessels. Subsequently, a 16G microwave ablation antenna (KY-2450A-1) was inserted into the center of the parathyroid adenoma under ultrasound guidance. Ablation was initiated at an output power of 20–30 W. The ablation power, duration, and antenna position were adjusted dynamically according to the distribution of hyperechoic microbubbles generated during thermal coagulation. Ablation was continued until the entire lesion was replaced by hyperechoic changes, after which the antenna was withdrawn. Immediately after the procedure, CEUS was repeated to assess treatment completeness. The absence of contrast enhancement within the ablation zone was considered indicative of complete ablation. Residual contrast enhancement was regarded as evidence of incomplete ablation, and additional ablation was performed immediately until complete non-enhancement was achieved. Throughout the procedure, blood pressure, heart rate, and peripheral oxygen saturation were continuously monitored. Ablation was temporarily interrupted if patients developed significant pain, hoarseness suggestive of recurrent laryngeal nerve irritation, or manifestations of a vagal reflex.

### 2.4 Parathyroidectomy procedure

Following successful induction of general anesthesia with endotracheal intubation, patients were placed in the supine position with mild neck extension. After routine sterilization and draping of the operative field, a transverse cervical incision approximately 6 cm in length was made one fingerbreadth above the sternal notch. The skin, subcutaneous tissue, and platysma were sequentially divided, and subplatysmal flaps were elevated superiorly to the inferior border of the thyroid cartilage, inferiorly to the sternal notch, and laterally to the anterior border of the sternocleidomastoid muscle. The cervical linea alba was divided, and dissection was continued to expose the thyroid capsule. Hyperfunctioning parathyroid glands were identified under direct visualization based on preoperative ultrasonographic localization and intraoperative palpation. Once identified, the affected gland was meticulously dissected while preserving the recurrent laryngeal nerve and branches of the inferior thyroid artery. Complete excision was performed for solitary parathyroid lesions, whereas subtotal parathyroidectomy was undertaken when multiglandular or bilateral disease was identified. Intraoperative frozen-section pathological examination was performed when necessary to confirm the diagnosis and assess the completeness of resection. After thorough irrigation of the operative field and meticulous hemostasis, a surgical drain was placed in the operative bed before layered wound closure. Patients received routine postoperative monitoring, including serial assessments of serum calcium and PTH levels, as well as evaluation of recurrent laryngeal nerve function.

### 2.5 Follow-up

All participants underwent laboratory evaluations at baseline and at multiple postoperative follow-up visits, including postoperative 1 day,7 days, 1 month, 3 months, and 6 months, with measurements of serum PTH, calcium, and phosphorus levels obtained at each time point. Thereafter, follow-up assessments were performed at 6-month intervals. Patients were followed for a minimum of 6 months or until the end of the study period. The duration of follow-up ranged from 6 to 66 months (median, 12 months) in the PTX group and from 6 to 24 months (median, 6 months) in the MWA group. Because the duration of follow-up differed substantially between groups, all primary comparative efficacy analyses and comparative time-to-event analyses were restricted to the common 6-month follow-up period. Observations obtained beyond 6 months were used only for descriptive analyses. Data collection was completed in January 2025.

### 2.6 End points

The primary endpoint of the study was biochemical cure, defined as normalization of both serum PTH and calcium levels maintained for at least 6 months after treatment. Treatment failure was classified as either persistent or recurrent disease. Persistent failure was defined as failure to achieve normalization of serum calcium and/or PTH levels within 6 months after treatment, whereas recurrent failure was defined as the reappearance of hypercalcemia and/or elevated PTH levels after initial biochemical cure. Secondary endpoints included longitudinal changes in serum PTH, calcium, and phosphorus levels following treatment, procedure-related complications, and technical success. Technical success was defined as complete absence of contrast enhancement within the treated lesion on contrast-enhanced ultrasound immediately after completion of the procedure.

### 2.7 Statistical analysis

All statistical analyses were performed using R software (version 4.1.0). Continuous variables were assessed for normality using the Kolmogorov–Smirnov test and for homogeneity of variance. Normally distributed variables with equal variances are presented as the mean ± standard deviation (SD) and were compared using the independent-samples t test. Non-normally distributed variables or variables with unequal variances are presented as the median (interquartile range [IQR]) and were compared using the Mann–Whitney U test. Categorical variables are expressed as frequencies (percentages) and were compared using the chi-square test. Baseline PTH was transformed using the base-2 logarithm because of its right-skewed distribution. The resulting odds ratio represents the change in the odds of treatment failure associated with each doubling of baseline PTH. Because treatment allocation was not randomized, covariate-balancing propensity score (CBPS)-derived inverse probability of treatment weighting (IPTW) was applied to reduce baseline imbalance between groups. Standardized mean differences (SMDs), rather than baseline P values, were used as the primary measure of covariate imbalance. Propensity scores were estimated using age, sex, log_2_-transformed PTH, serum calcium, renal function, nephrolithiasis, maximum gland diameter, and number of localized glands. Covariate balance after weighting was assessed using standardized mean differences, with an absolute SMD <0.10 indicating adequate balance.

Longitudinal trajectories of serum PTH, calcium, and phosphorus were analyzed using IPTW-weighted linear mixed-effects models. Treatment modality, categorical follow-up time, and the treatment-by-time interaction were included as fixed effects. A patient-specific random intercept was incorporated to account for intra-individual correlation from repeated measurements. The treatment-by-time interaction was prespecified as the primary parameter of interest for evaluating whether biochemical trajectories differed between PTX and MWA. Estimated marginal means with 95% confidence intervals were calculated at each follow-up time point. All longitudinal analyses were restricted to the common 6-month follow-up period to minimize bias related to unequal follow-up duration.

After CBPS-derived IPTW adjustment, treatment outcomes were compared between the PTX and MWA groups. Weighted logistic regression models with robust standard errors were used to estimate the association between treatment modality and binary outcomes, including overall treatment failure, persistent failure, post-treatment hypocalcemia, and any recorded complication. Overall treatment failure was defined as persistent or recurrent biochemical failure after intervention. Results were presented as IPTW-weighted risks, odds ratios (ORs), risk differences (RDs), and corresponding 95% confidence intervals (CIs). Because follow-up duration differed between treatment groups, time-to-event analyses were restricted to the common 6-month follow-up period. Failure-free survival was defined as the time from treatment initiation to the first occurrence of recorded treatment failure. Patients without treatment failure were censored at the last available follow-up assessment. IPTW-weighted Kaplan–Meier curves were generated to estimate failure-free probabilities over time. An IPTW-weighted Cox proportional hazards model was performed as an exploratory analysis to evaluate the association between treatment modality and time to first recorded treatment failure. Given the limited number of failure events, hazard ratios (HRs) with 95% CIs were emphasized.

To identify factors associated with treatment failure, univariable and multivariable Firth penalized logistic regression models were performed. Firth correction was applied to reduce small-sample bias and potential separation issues due to the limited number of treatment failure events. Potential multicollinearity among covariates included in multivariable regression models was assessed using variance inflation factors (VIFs). A VIF >5 was considered indicative of clinically relevant collinearity. The results are reported as ORs with corresponding 95% confidence intervals (CIs). A sensitivity power analysis was performed using unequal-group two-sample proportion tests. Based on 127 PTX and 46 MWA patients, a two-sided α of 0.05, and a reference cure rate of 75.6%, we estimated power for a 10-percentage-point difference and the minimum detectable difference at 80% power. Missing data were handled using multiple imputation. All statistical tests were two-sided, and a P value < 0.05 was considered statistically significant. The significance level was set at α = 0.05.

## 3 Results

### 3.1 Participant characteristics

The study cohort included 173 patients, comprising 127 patients who underwent PTX and 46 patients who underwent MWA. Baseline characteristics of the included patients are summarized in **Tables 1**, **2**. Before IPTW adjustment, clinically relevant differences were observed between the treatment groups, particularly in log_2_-transformed baseline PTH levels (absolute SMD, 0.429), nephrolithiasis (absolute SMD, 0.385), maximum gland diameter (absolute SMD, 0.380), serum calcium levels (absolute SMD, 0.220), and the number of localized glands (absolute SMD, 0.209). After CBPS-derived IPTW adjustment, all prespecified covariates achieved adequate balance between groups, with absolute standardized mean differences (SMDs) reduced to <0.01 (**Table 3**; **Figure 1**). The stabilized weights showed acceptable distributions, ranging from 1.02 to 5.88 in the PTX group and from 1.46 to 11.81 in the MWA group. The effective sample sizes after weighting were 113.0 and 35.0 for the PTX and MWA groups, respectively, indicating that the weighting procedure preserved a substantial proportion of the available information (**Supplementary Table 1**).

**Table 1:** Baseline clinical and biochemical characteristics of patients with primary hyperparathyroidism (n=173).

Characteristic	Total (n = 173)	PTX (n = 127)	MWA (n = 46)	P
Age (y)	56.00 (47.00, 62.00)	55.00 (46.50, 61.50)	57.50 (47.25, 66.25)	0.336
Bmi (kg/m2)	24.44 (22.04, 26.67)	24.45 (21.88, 26.74)	24.13 (22.19, 26.36)	0.745
Female, n (%)	135 (78.03)	99 (77.95)	36 (78.26)	0.966
Serum PTH (pg/ml)	143.40 (105.00, 346.70)	152.00 (107.65, 401.38)	130.50 (103.50, 202.75)	0.043
Serum calcium (mmol/L)	2.71 (2.57, 2.86)	2.71 (2.57, 2.88)	2.69 (2.56, 2.77)	0.136
Serum phosphorus (mmol/L)	0.82 ± 0.18	0.82 ± 0.17	0.82 ± 0.21	0.915
Serum magnesium (mmol/L)	0.89 (0.84, 0.95)	0.90 (0.84, 0.95)	0.89 (0.86, 0.96)	0.851
25(OH)D3 (ng/ml)	9.20 (6.60, 14.30)	9.10 (6.60, 14.80)	9.80 (8.03, 11.90)	0.997
24-hour urinary calcium (mmol/24h)	0.46 ± 0.66	0.48 ± 0.69	0.42 ± 0.59	0.641
ALP (U/L)	97.00 (74.00, 142.00)	100.00 (74.50, 149.00)	88.50 (71.00, 115.00)	0.131
Cystatin C (mg/L)	0.82 (0.71, 1.00)	0.80 (0.70, 0.96)	0.90 (0.73, 1.20)	0.029
Uric acid (µmol/L)	332.00 (272.00, 396.00)	324.00 (269.00, 383.50)	366.50 (303.00, 444.50)	0.061
Serum osteocalcin (ng/ml)	52.60 (22.89, 64.15)	52.60 (22.89, 64.15)	52.60 (11.24, 64.15)	0.773
Maximum diameter (cm)	1.60 (1.20, 2.50)	1.70 (1.30, 2.55)	1.55 (1.00, 2.08)	0.078
Cure, n (%)	131 (75.72)	96 (75.59)	35 (76.09)	1.000

Data are presented as mean ± SD, median (interquartile range), or number (percentage), as appropriate. P values indicate comparisons between PTX and MWA. Statistical significance was defined as P <.05.

BMI, body mass index; PTH, parathyroid hormone; 25(OH)D3, 25-hydroxyvitamin D3; ALP, alkaline phosphatase; PTX, parathyroidectomy; MWA, microwave ablation. Statistical significance was defined as P <.05.

**Table 2:** Clinical manifestations, comorbidities, and adenoma characteristics of patients.

Characteristic	Total (n = 173)	PTX (n = 127)	MWA (n = 46)	P
The number of adenomas, n (%)				0.240
1	160 (92.49)	119 (93.70)	41 (89.13)	
2	10 (5.78)	7 (5.51)	3 (6.52)	
3	3 (1.73)	1 (0.79)	2 (4.35)	
Renal insufficiency, n (%)	51 (29.48)	30 (23.62)	21 (45.65)	0.005
Hypertension, n (%)	60 (34.68)	42 (33.07)	18 (39.13)	0.459
Diabetes, n (%)	31 (17.92)	19 (14.96)	12 (26.09)	0.092
Kidney stone, n (%)	84 (48.55)	68 (53.54)	16 (34.78)	0.029
Fracture, n (%)	8 (4.62)	7 (5.51)	1 (2.17)	0.607
Fatigue, n (%)	18 (10.40)	15 (11.81)	3 (6.52)	0.469
Ostealgia, n (%)	31 (17.92)	23 (18.11)	8 (17.39)	0.913

Adenoma distribution comorbidities, and clinical symptoms are summarized according to study groups. Data are presented as number (percentage).

PTX, parathyroidectomy; MWA, microwave ablation. Statistical significance was defined as P <.05.

**Table 3:** Baseline covariate balance before and after IPTW adjustment.

Covariate	Absolute SMD before IPTW	Absolute SMD after IPTW
Age	0.199	<0.01
Female	0.007	<0.01
Log_2_-transforme_d_ PTH	0.429	<0.01
Serum calcium	0.220	<0.01
eGFR	0.018	<0.01
Nephrolithiasis	0.385	<0.01
Maximum gland diameter	0.380	<0.01
Number of localized glands	0.209	<0.01

Absolute standardized mean differences were used to assess covariate balance between the PTX and MWA groups. Inverse probability of treatment weights were derived using the covariate-balancing propensity score and targeted the average treatment effect. An absolute SMD <0.10 was considered indicative of acceptable balance.

CBPS, covariate-balancing propensity score; eGFR, estimated glomerular filtration rate; IPTW, inverse probability of treatment weighting; PTH, parathyroid hormone; SMD, standardized mean difference.

**Figure 1:**
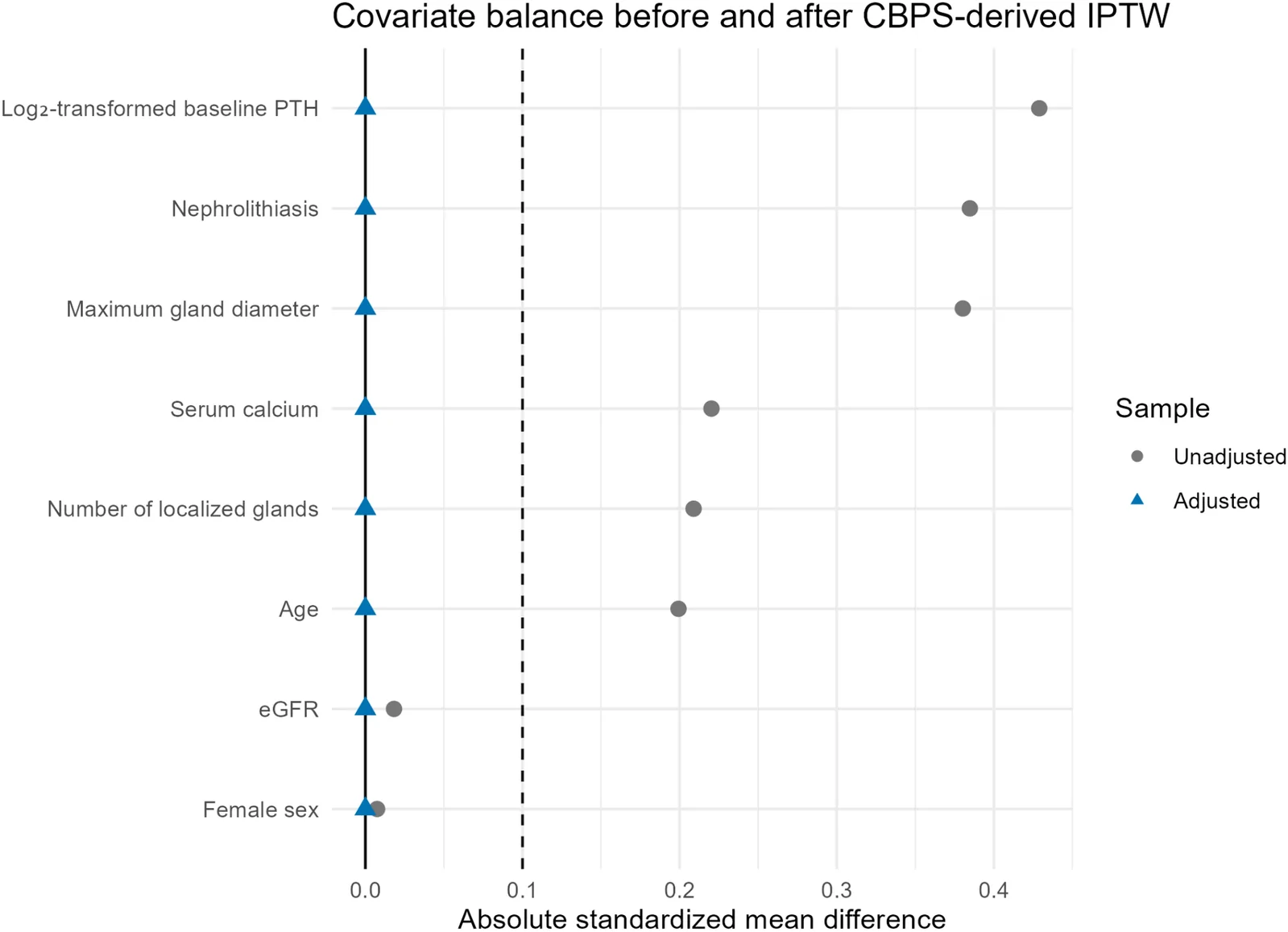
Covariate balance before and after covariate-balancing propensity score–derived inverse probability of treatment weighting. Gray circles indicate unadjusted absolute standardized mean differences, and blue triangles indicate weighted absolute standardized mean differences for the prespecified baseline covariates. The dashed vertical line at 0.10 denotes the threshold for acceptable covariate balance.

### 3.2 Therapeutic efficacy

After CBPS-derived IPTW, longitudinal changes in serum PTH, calcium, and phosphorus were assessed using IPTW-weighted linear mixed-effects models. Compared with baseline, serum PTH levels decreased markedly after intervention and remained substantially lower throughout follow-up in both groups. However, a significant treatment-by-time interaction was observed for PTH trajectories (P< 0.001), indicating that the temporal patterns of PTH decline differed between PTX and MWA (**Figure 2**). Serum calcium concentrations demonstrated an early decline after treatment, followed by gradual recovery toward the normal range during follow-up in both groups. The treatment-by-time interaction for calcium was not statistically significant (P>0.05), suggesting comparable patterns of calcium recovery after adjustment (**Figure 3**). Serum phosphorus levels also showed comparable longitudinal changes between groups, with no significant treatment-by-time interaction detected (P>0.05) (**Figure 4**). Detailed IPTW-weighted estimated marginal means of biochemical parameters at each follow-up time point are provided in **Supplementary Table 2**.

**Figure 2:**
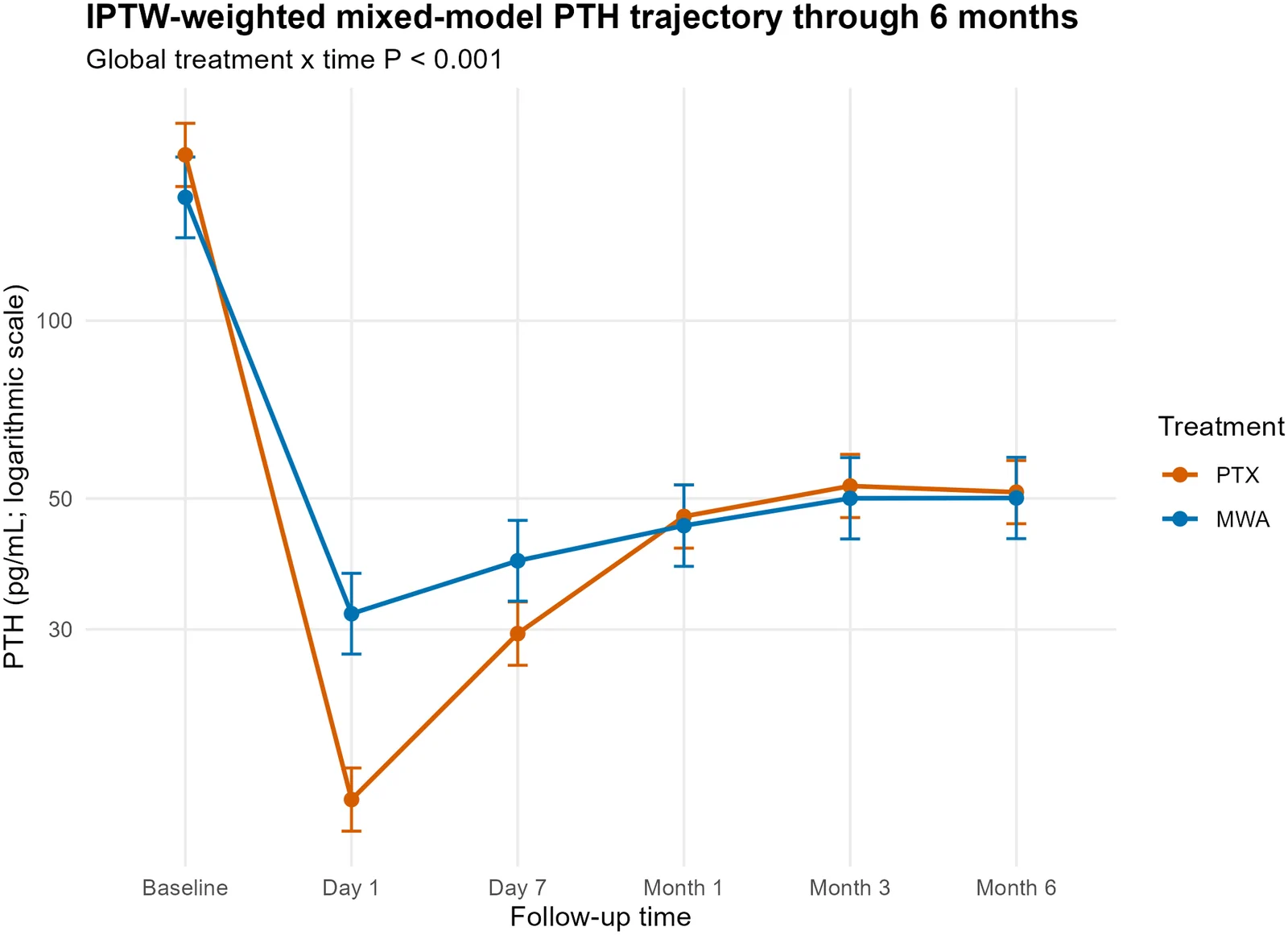
Inverse probability of treatment–weighted longitudinal trajectory of serum parathyroid hormone during the first 6 months after treatment. Points represent estimated marginal means from the weighted linear mixed-effects model, back-transformed to the original PTH scale, and error bars represent 95% confidence intervals. PTH was modeled on the log_2_ scale, and the back-transformed estimates are displayed in pg/mL on a logarithmic y-axis.

**Figure 3:**
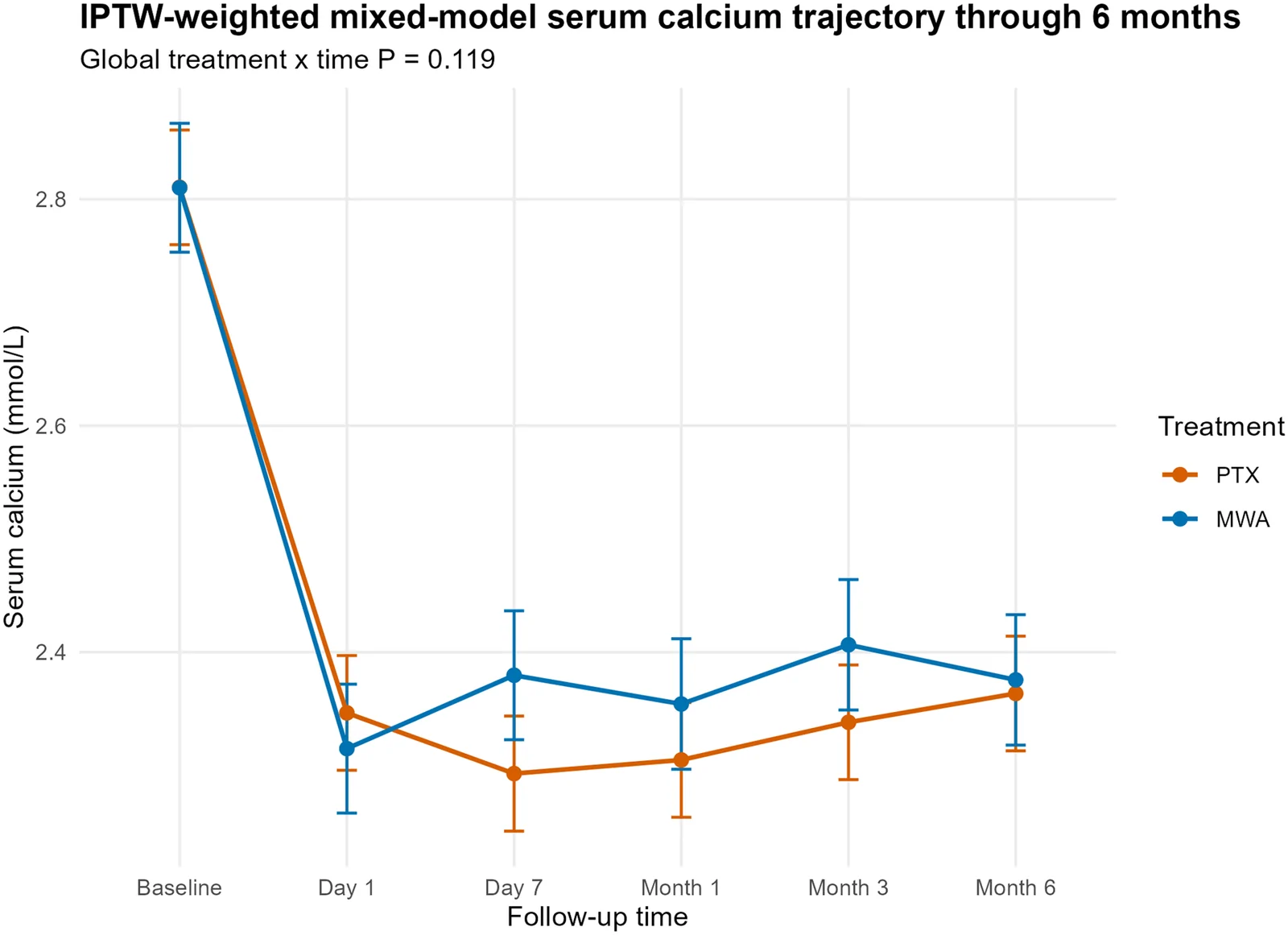
Inverse probability of treatment–weighted longitudinal trajectory of serum calcium during the first 6 months after treatment.

**Figure 4:**
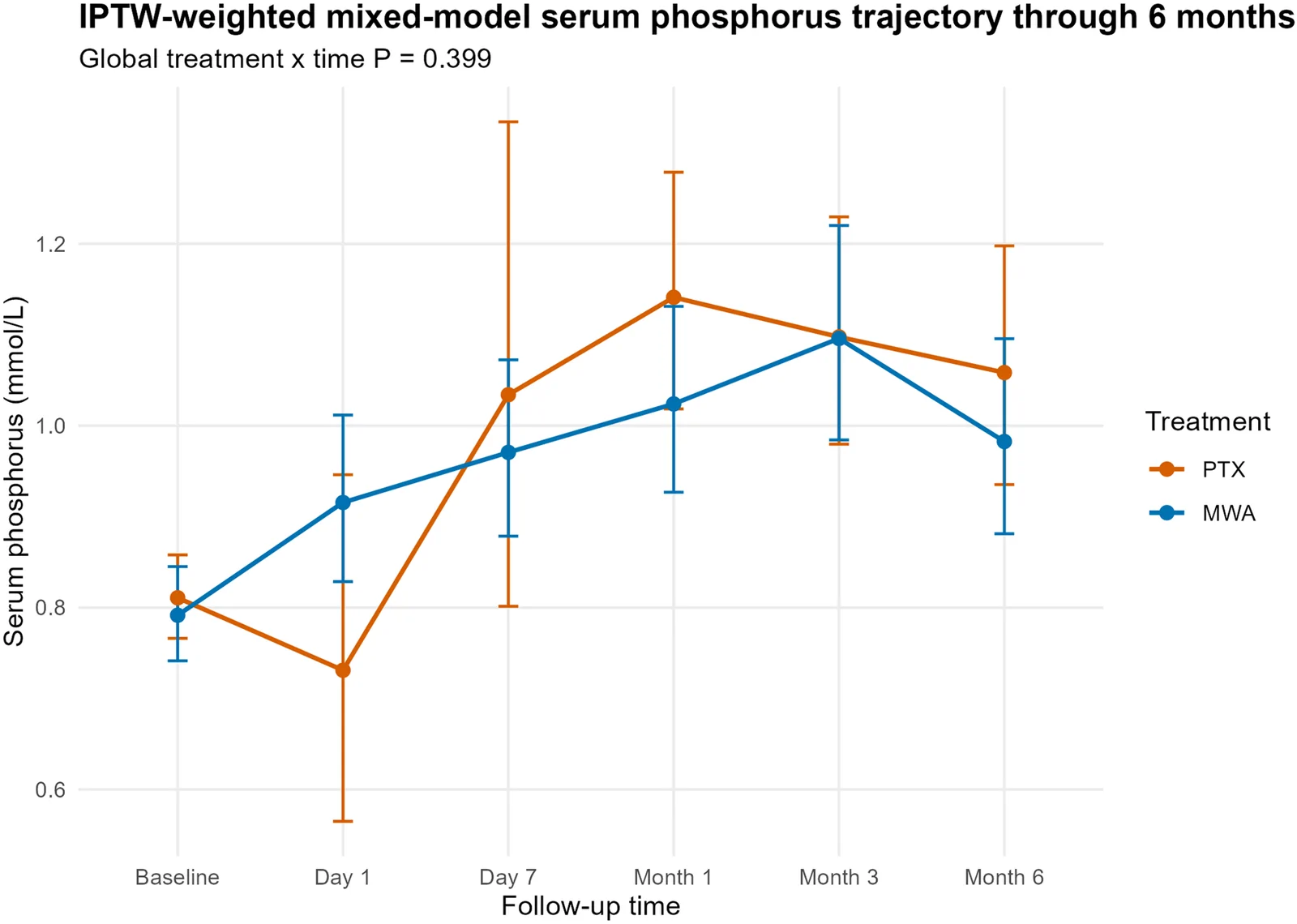
Inverse probability of treatment–weighted longitudinal trajectory of serum phosphorus during the first 6 months after treatment.

After CBPS-derived IPTW adjustment, treatment outcomes were compared between the PTX and MWA groups (**Table 4**). The weighted risk of overall treatment failure was 23.8% in the PTX group and 35.9% in the MWA group. The difference was not statistically significant (OR, 1.80; 95% CI, 0.74–4.39; P>0.05). Similarly, the weighted risk of persistent failure was 22.3% for PTX and 33.9% for MWA. No statistically significant difference was observed between treatment groups (OR, 1.79; 95% CI, 0.71–4.54; P>0.05). The corresponding absolute risk differences were 12.1 percentage points for overall treatment failure (95% CI, −7.3 to 31.6) and 11.6 percentage points for persistent failure (95% CI, −8.1 to 31.3) (**Supplementary Table 3**).

**Table 4:** IPTW-weighted comparison of treatment efficacy and safety outcomes between PTX and MWA.

Outcome	PTX weighted risk	MWA weighted risk	OR (95% CI)	P
Overall treatment failure	23.8%	35.9%	1.80 (0.74 to 4.39)	0.198
Persistent failure	22.3%	33.9%	1.79 (0.71 to 4.54)	0.220
Hypocalcemia	9.2%	15.5%	1.81 (0.50 to 6.56)	0.364

Weighted risks are marginal risks estimated for each treatment group. Effect estimates are odds ratios with robust 95% confidence intervals obtained from IPTW-weighted logistic regression, with PTX as the reference group. P values are two-sided.

IPTW, inverse probability of treatment weighting; MWA, microwave ablation; PTX, parathyroidectomy; OR, odds ratio; HR, hazard ratio; CI, confidence interval.

As an exploratory analysis, IPTW-weighted time-to-event analyses were restricted to the common 6-month follow-up period. The weighted Kaplan–Meier analysis demonstrated that failure-free probability gradually declined over time in both groups (**Figure 5**). The IPTW-weighted Cox proportional hazards model showed no statistically significant difference in the hazard of first recorded treatment failure between MWA and PTX (HR, 1.73; 95% CI, 0.82–3.64; P>0.05).

**Figure 5:**
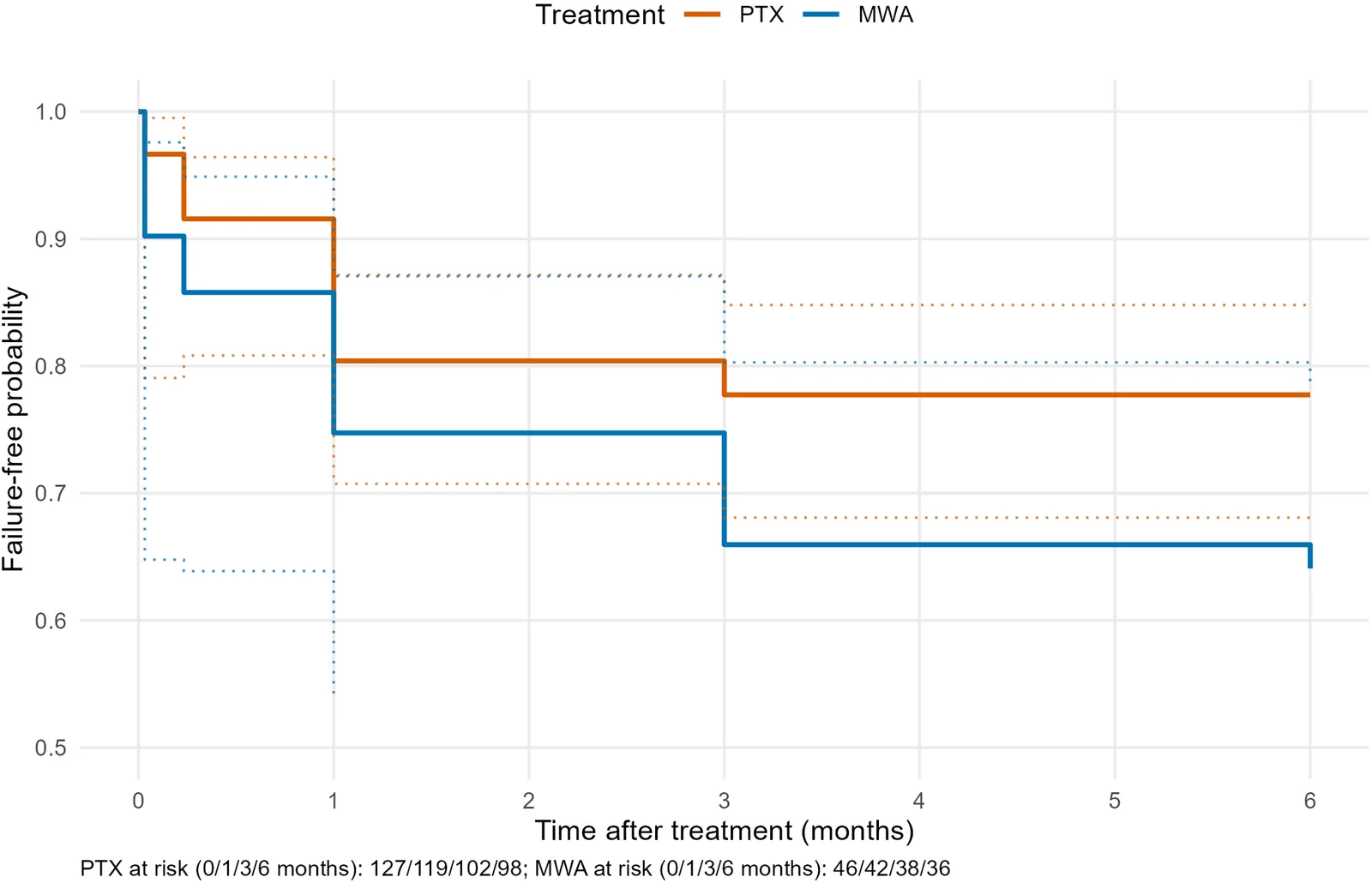
Inverse probability of treatment–weighted Kaplan–Meier curves for failure-free probability during the common 6-month follow-up period. Dotted lines represent 95% confidence intervals.

In the sensitivity power analysis, the statistical power to detect a 10-percentage-point higher or lower biochemical cure rate in the MWA group was 31.6% and 24.9%, respectively. At 80% power, the minimum detectable absolute difference was 17.0 percentage points for a higher MWA cure rate and 22.8 percentage points for a lower MWA cure rate. These results indicated limited power to detect moderate between-group differences.

### 3.3 Safety outcomes

Immediate postprocedural contrast-enhanced ultrasound demonstrated complete non-enhancement of all treated lesions after microwave ablation, corresponding to a technical success rate of 100%. The primary complications observed were hypocalcemia and transient voice changes. The weighted risks of post-treatment hypocalcemia were 9.2% in the PTX group and 15.5% in the MWA group, without a statistically significant difference (OR, 1.81; 95% CI, 0.50–6.56; P >0.05). Mild transient hypocalcemia accompanied by perioral and extremity paresthesia occurred in 17 patients (9.82%) within 1–3 days after treatment, including 13 patients (10.23%) in the PTX group and 4 patients (8.69%) in the MWA group. Symptoms resolved within one week following oral supplementation with calcitriol and calcium. No significant difference in the incidence of hypocalcemia was observed between the two treatment groups (P > 0.05). Transient voice changes occurred in one patient (2.17%) in the MWA group and resolved spontaneously within 1 month without specific treatment. No other procedure-related complications were observed.

### 3.4 Risk factors associated with treatment failure

Predictors of treatment failure were evaluated using Firth penalized logistic regression analyses. No evidence of clinically relevant multicollinearity was observed among covariates included in the multivariable model, with VIF values ranging from 1.02 to 1.90, all below the prespecified threshold of 5 (**Supplementary Table 4**). In univariable analyses, higher baseline PTH levels, higher serum calcium concentrations, renal insufficiency, lower serum phosphorus concentrations, and larger maximum gland diameter were associated with treatment failure, whereas treatment modality was not significantly associated with outcome (**Supplementary Table 5**; **Figure 6**). In the multivariable Firth penalized logistic regression model, baseline PTH remained an independent predictor of treatment failure. Each doubling of baseline PTH was associated with approximately twofold higher odds of 6-month biochemical treatment failure (OR, 2.02; 95% CI, 1.39–4.09; P <no><</no> 0.05). In contrast, treatment modality (MWA vs PTX) was not independently associated with treatment failure after adjustment (OR, 2.25; 95% CI, 0.78–5.12; P>0.05) (**Supplementary Table 5**; **Figure 7**).

**Figure 6:**
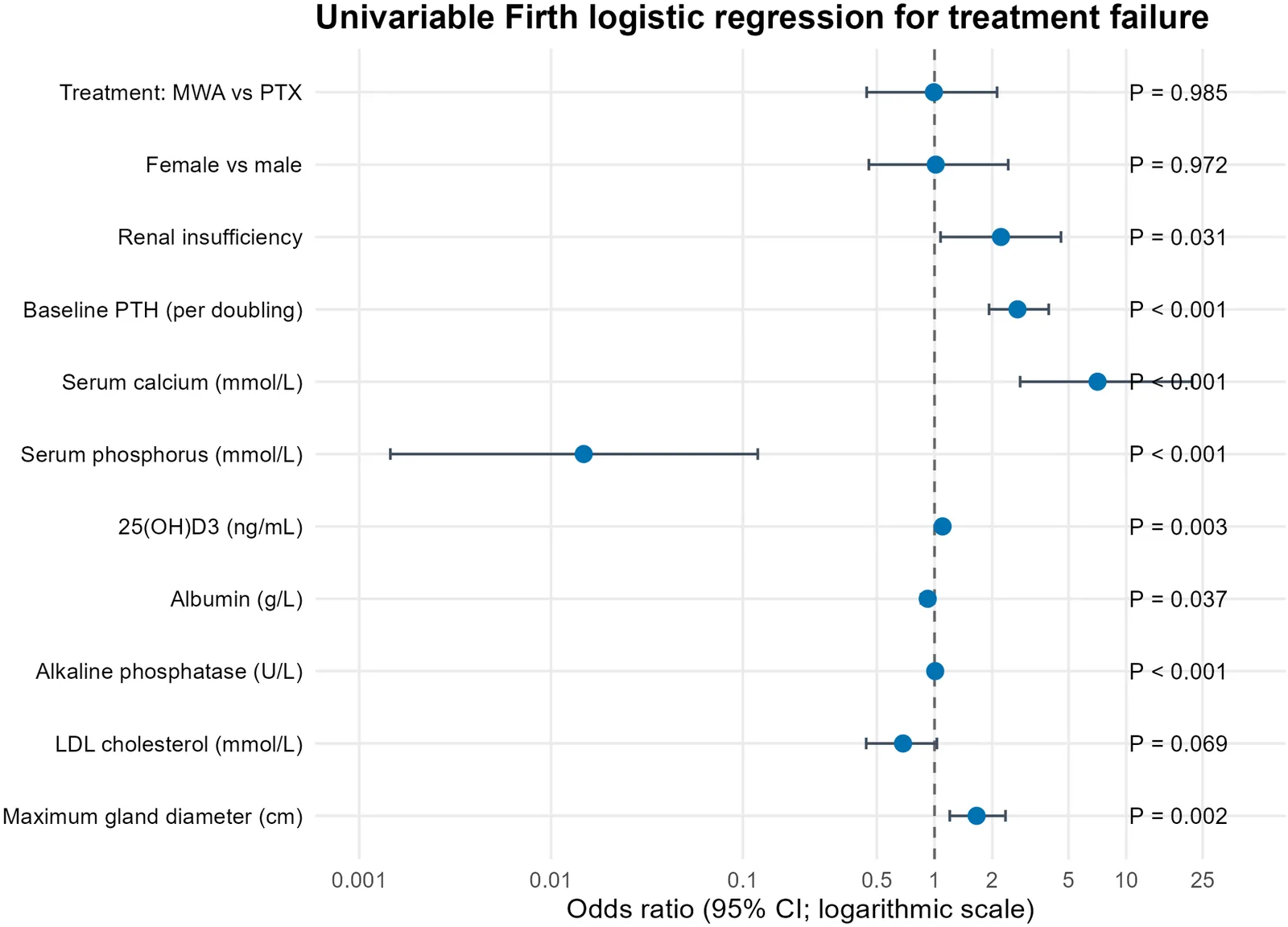
Univariable Firth penalized logistic regression analysis of factors associated with biochemical treatment failure. Points represent odds ratios, and horizontal bars represent profile penalized-likelihood 95% confidence intervals. The vertical reference line denotes an odds ratio of 1.

**Figure 7:**
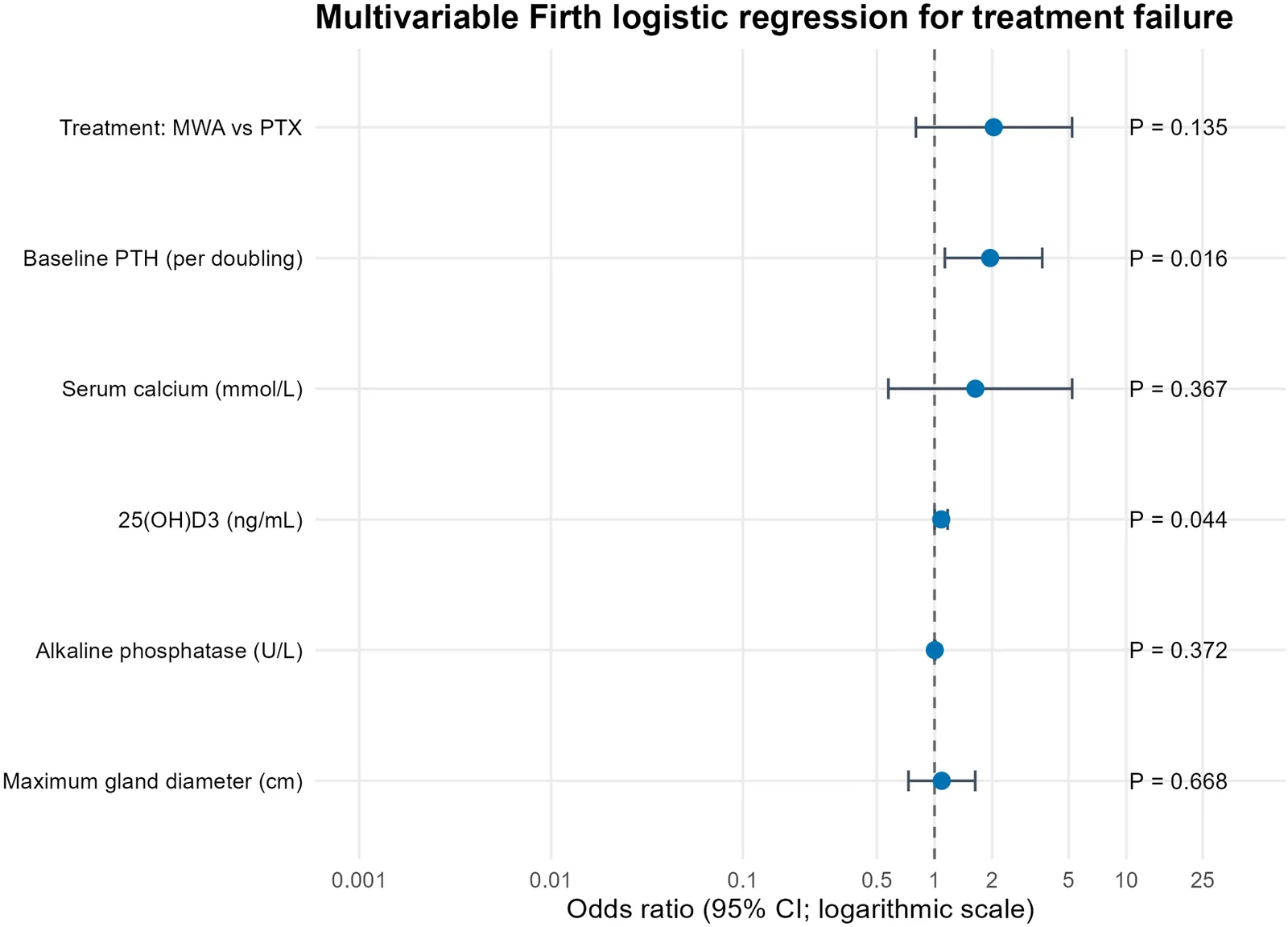
Multivariable Firth penalized logistic regression analysis of factors associated with biochemical treatment failure.

## 4 Discussion

In recent years, MWA, with its advantages of precise image guidance, minimal invasiveness, and rapid recovery, has emerged as a promising alternative therapeutic strategy for PHPT (14–16).

In this retrospective cohort study of patients with PHPT, both MWA and PTX were associated with substantial short-term improvements in serum PTH, calcium, and phosphorus levels. After measured baseline differences were balanced using CBPS-IPTW, no statistically significant between-group difference was detected in biochemical treatment failure. Nevertheless, the weighted point estimate suggested a numerically higher failure risk after MWA, with an absolute risk difference of 12.1 percentage points and an odds ratio of 1.80, and the corresponding confidence intervals were wide. The exploratory 6-month time-to-event analysis showed a similar pattern. These findings therefore indicate that the study did not establish a definitive difference between the two treatments.

Previous comparative studies have also suggested that thermal ablation may achieve biochemical outcomes approaching those of surgery in appropriately selected patients. Liu et al. used propensity score matching and reported cure rates of 82.1% after MWA and 89.3% after surgical resection (17). Wei et al. subsequently compared 87 matched patients in each treatment group and found no statistically significant differences in clinical cure, persistent disease, recurrence, or major complications during a median follow-up of 28.5 months (18). In addition, a prospective multicenter study including 132 patients treated with MWA or radiofrequency ablation reported an overall cure rate of 80.3% (19). The present findings are broadly consistent with this developing evidence base. Compared with previous studies, the present study used CBPS-IPTW to retain the full eligible cohort, restricted all principal comparisons to a common 6-month observation window, and evaluated repeated biochemical measurements through formal treatment-by-time interaction tests rather than relying exclusively on cure rates at a single time point. These methodological features constitute the principal contribution of this study.

The longitudinal analyses provide additional insight into the biochemical response to treatment. Although both MWA and PTX produced marked reductions in PTH, the significant treatment-by-time interaction indicated that the timing or magnitude of the early PTH response differed between the two interventions. PTX directly removes the identified hyperfunctioning tissue, whereas MWA destroys the target lesion *in situ* through thermal coagulation, followed by progressive evolution and resorption of the ablation zone. These distinct procedural mechanisms may plausibly result in different early PTH trajectories (20–22). However, this explanation remains mechanistic and cannot be directly established from the present observational data. In contrast, no significant treatment-by-time interactions were observed for serum calcium or phosphorus, suggesting that the difference in PTH trajectories did not translate into clearly distinguishable short-term patterns of calcium–phosphate recovery. Serial biochemical monitoring therefore remains important after either intervention, particularly during the early post-treatment period (23, 24).

The proportion of patients who met the biochemical cure definition in the present cohort was lower than the cure rates commonly reported after PTX and lower than those in some thermal ablation studies. Parathyroidectomy performed by experienced surgeons generally achieves cure rates exceeding 90%, whereas conventional surgical cure is primarily defined by sustained eucalcemia for more than 6 months (25, 26). By contrast, the present study applied a more stringent study-specific endpoint requiring both serum calcium and PTH to be within their respective reference ranges at the 6-month assessment. Consequently, patients with normal calcium but persistently elevated PTH were classified as having biochemical treatment failure (27). This stricter definition probably contributed to the lower apparent cure rate compared with studies relying primarily on normalization of serum calcium. Thermal ablation studies using combined normalization of PTH and calcium have reported cure rates of approximately 75%–85%, although differences in patient selection, lesion burden, follow-up completeness, outcome definitions, and retreatment strategies limit direct comparisons (19, 28–30).

The discrepancy between the 100% immediate technical success rate after MWA and the substantially lower biochemical cure rate also requires careful interpretation. Complete non-enhancement on immediate contrast-enhanced ultrasonography confirms the absence of detectable perfusion within the treated target lesion but does not demonstrate that all hyperfunctioning parathyroid tissue has been eliminated. Biochemical failure may result from incomplete treatment of the lesion margin, residual viable tissue not detected on immediate imaging, inaccurate lesion localization, or an additional hyperfunctioning gland. Previous research has identified larger parathyroid nodule volume as an independent factor associated with a lower probability of cure after MWA, supporting the importance of lesion burden and complete ablation coverage (25, 31). Furthermore, neither cervical ultrasonography nor sestamibi scintigraphy can completely exclude occult multiglandular disease (32, 33). Technical success should therefore be interpreted as a procedural endpoint rather than a surrogate for biochemical cure.

The absence of routine intraoperative PTH monitoring may also have contributed to the lower cure rate, particularly in patients with occult multiglandular disease or discordant localization findings. Intraoperative PTH monitoring can provide immediate evidence regarding the adequacy of resection during focused parathyroidectomy and is recommended when minimally invasive parathyroidectomy is performed using a reliable monitoring protocol (34–36). Its role in image-guided thermal ablation is less well established, because an immediate decline in circulating PTH may not fully reflect the subsequent biological evolution of thermally treated tissue (37). Future comparative studies should therefore develop and evaluate procedure-specific peri-interventional PTH monitoring protocols.

Baseline PTH was independently associated with biochemical treatment failure in the present study. Because PTH was modeled on the log_2_ scale, the odds ratio of 2.02 indicates that each doubling of baseline PTH was associated with approximately twice the odds of treatment failure. This clinically interpretable association suggests that markedly elevated PTH may reflect a greater functional parathyroid tissue burden, more severe secretory activity, a larger lesion, or unrecognized multiglandular disease (18, 38, 39). Collectively, these findings suggest that baseline disease burden and lesion extent may be important determinants of biochemical response.

Treatment modality was not statistically significantly associated with treatment failure in the multivariable Firth model. However, the point estimate for MWA versus PTX remained above unity, and its confidence interval was wide. The sensitivity power analysis further showed that the study had only 31.6% and 24.9% power to detect a 10-percentage-point higher or lower cure rate in the MWA group, respectively. At 80% power, the minimum detectable absolute differences were 17.0 percentage points for a higher MWA cure rate and 22.8 percentage points for a lower MWA cure rate. Therefore, the absence of statistical significance does not exclude clinically meaningful differences between the interventions.

This study has several methodological strengths. First, CBPS-IPTW directly addressed the substantial baseline differences caused by nonrandom treatment selection and achieved close balance across all prespecified measured covariates. Second, restricting the comparative analyses to the common 6-month observation period reduced bias arising from the substantially longer follow-up available in the PTX group. Third, the repeated-measures analysis evaluated complete biochemical trajectories and formally tested treatment-by-time interactions, thereby providing information beyond a single fixed-time cure assessment. Fourth, the stringent dual-normalization endpoint and the Firth penalized prognostic model enabled a transparent evaluation of incomplete biochemical response in a relatively small cohort. The novelty of the present study therefore lies in its adjusted short-term comparison and characterization of potentially different early biochemical response patterns.

Several limitations should also be acknowledged. First, despite the close balance of measured covariates after IPTW, the retrospective single-center design remains vulnerable to residual and unmeasured confounding. Treatment selection may have been influenced by patient frailty, preference, lesion location or composition, imaging confidence, operator experience, perceived surgical risk, and other variables that were not fully captured in the propensity score model (40). Second, only 46 patients underwent MWA, and the effective sample size was further reduced after weighting; the study was therefore unable to exclude clinically meaningful between-group differences. Third, restricting the analysis to a common 6-month window improved the fairness of the short-term comparison but prevented an adequate assessment of long-term durability and recurrence. Fourth, the stringent biochemical endpoint enhanced the detection of incomplete recovery but limited direct comparison with studies defining cure solely according to sustained eucalcemia. Fifth, occult multiglandular disease, incomplete ablation coverage, and the absence of routine intraoperative or peri-interventional PTH monitoring may have influenced the cure estimates.

Future research should use prospective multicenter designs with balanced and sufficiently long follow-up and standardized biochemical and clinical outcome definitions. When the objective is to establish MWA as an alternative to PTX, an acceptable non-inferiority margin should be specified in advance and used to determine the required sample size. Future studies should also include more comprehensive assessment of multiglandular disease, systematic recording of lesion volume and composition, standardized evaluation of ablation margins, measures of operator experience, and procedure-specific PTH monitoring protocols. Long-term recurrence, retreatment, skeletal and renal outcomes, quality of life, and patient treatment preferences should also be evaluated.

In conclusion, both MWA and PTX were associated with substantial short-term biochemical improvement in selected patients with PHPT. After CBPS-IPTW adjustment, no statistically significant difference in 6-month treatment failure was detected; however, the wide confidence intervals and limited statistical power did not support conclusions of equivalence or non-inferiority. The distinct early parathyroid hormone trajectories and the prognostic relevance of baseline PTH provide additional insight into treatment response and may support more individualized patient assessment. Microwave ablation may be considered a minimally invasive option for carefully selected patients, but its long-term comparative effectiveness requires confirmation in adequately powered prospective studies with balanced follow-up.

## Data Availability

The raw data supporting the conclusions of this article will be made available by the authors, without undue reservation.
