# Supplementary material for: Short-term biochemical outcomes after microwave ablation versus parathyroidectomy for primary hyperparathyroidism: an IPTW analysis
Source: Front Endocrinol (Lausanne). 2026 Sep 16;17:1921824. doi: 10.3389/fendo.2026.1921824 (PMC13623593; doi:10.3389/fendo.2026.1921824)
Supplement: Supplementary file 1 [file Table1.docx]

Supplementary Materials

Supplementary Table 1. IPTW weight diagnostics

| Variable | PTX | MWA |
| --- | --- | --- |
| Original sample size | 127 | 46 |
| Effective sample size after IPTW | 113 | 35 |
| Mean stabilized weight | 1.00 | 1.00 |
| Minimum weight | 1.02 | 1.46 |
| Maximum weight | 5.88 | 11.81 |

Abbreviations: IPTW, inverse probability of treatment weighting; MWA, microwave ablation; PTX, parathyroidectomy

Supplementary Table 2. IPTW-weighted estimated marginal means of biochemical parameters during the 6-month follow-up after parathyroidectomy and microwave ablation

| Time point | PTX (95% CI) | MWA (95% CI) | P（PTX vs baseline ） | P（MWA vs baseline） |
| --- | --- | --- | --- | --- |
| Serum PTH (geometric mean, pg/mL) |  |  |  |  |
| Baseline | 190.9 (168.8–216.0) | 161.8 (138.2–189.4) | - | - |
| Day 1 | 15.5 (13.7–17.5) | 31.9 (27.3–37.3) | <0.001 | <0.001 |
| Day 7 | 29.5 (26.1–33.4) | 39.2 (33.5–45.9) | <0.001 | <0.001 |
| Month 1 | 46.6 (41.2–52.7) | 45.0 (38.4–52.7) | <0.001 | <0.001 |
| Month 3 | 52.5 (46.4–59.4) | 50.0 (42.7–58.6) | <0.001 | <0.001 |
| Month 6 | 51.2 (45.3–58.0) | 50.1 (42.7–58.7) | <0.001 | <0.001 |
| Serum calcium (mmol/L) |  |  |  |  |
| Baseline | 2.810 (2.760–2.861) | 2.810 (2.753–2.867) | - | - |
| Day 1 | 2.346 (2.296–2.397) | 2.315 (2.258–2.372) | <0.001 | <0.001 |
| Day 7 | 2.293 (2.242–2.344) | 2.380 (2.323–2.437) | <0.001 | <0.001 |
| Month 1 | 2.305 (2.254–2.356) | 2.354 (2.297–2.412) | <0.001 | <0.001 |
| Month 3 | 2.338 (2.287–2.389) | 2.407 (2.349–2.464) | <0.001 | <0.001 |
| Month 6 | 2.364 (2.313–2.414) | 2.376 (2.318–2.433) | <0.001 | <0.001 |
| Serum phosphorus (mmol/L) |  |  |  |  |
| Baseline | 0.811 (0.766–0.858) | 0.792 (0.741–0.845) | - | - |
| Day 1 | 0.731 (0.565–0.946) | 0.916 (0.828–1.012) | 0.439 | 0.470 |
| Day 7 | 1.034 (0.801–1.334) | 0.971 (0.879–1.072) | 0.654 | 0.412 |
| Month 1 | 1.141 (1.018–1.279) | 1.024 (0.927–1.131) | <0.001 | 0.337 |
| Month 3 | 1.098 (0.980–1.230) | 1.096 (0.984–1.220) | 0.086 | 0.189 |
| Month 6 | 1.058 (0.935–1.198) | 0.983 (0.881–1.096) | 0.372 | 0.575 |

Abbreviations: PTX, parathyroidectomy; MWA, microwave ablation; PTH, parathyroid hormone; CI, confidence interval；PTH values are geometric means; calcium and phosphorus values are arithmetic means.

Supplementary Table 3. IPTW-weighted absolute risk differences for efficacy and safety outcomes between MWA and PTX

| Outcome | PTX weighted risk (%) | MWA weighted risk (%) | RD | 95% CI | P value |
| --- | --- | --- | --- | --- | --- |
| Overall treatment failure | 23.8 | 35.9 | +12.1 | −7.3 to 31.6 | 0.221 |
| Persistent failure | 22.3 | 33.9 | +11.6 | −8.1 to 31.3 | 0.247 |
| Hypocalcemia | 9.2 | 15.5 | +6.3 | −9.5 to 22.1 | 0.432 |

Abbreviations: IPTW, inverse probability of treatment weighting; MWA, microwave ablation; PTX, parathyroidectomy; RD, risk difference; CI, confidence interval; Risk differences were estimated from CBPS-derived IPTW-weighted models and represent the absolute difference in outcome probability between MWA and PTX groups. Positive values indicate a higher estimated risk in the MWA group.

Supplementary Table 4. Variance inflation factors for covariates included in the IPTW-adjusted multivariable Firth logistic regression model

| Covariate | Weighted VIF |
| --- | --- |
| Treatment |  |
| PTX | - |
| MWA | 1.07 |
| Log baseline PTH | 1.90 |
| Serum calcium | 1.22 |
| 25(OH)D3 | 1.02 |
| Alkaline phosphatase | 1.51 |
| Maximum gland diameter | 1.31 |

Abbreviations: MWA, microwave ablation; PTX, parathyroidectomy; PTH, parathyroid hormone;

Supplementary Table 5. IPTW-adjusted Firth penalized logistic regression analyses for predictors of treatment failure

| Variable | Univariable OR (95% CI) | P | Multivariable OR (95% CI) | P |
| --- | --- | --- | --- | --- |
| Treatment |  |  |  |  |
| PTX | Reference | - | Reference | - |
| MWA | 1.05 (0.42–2.65) | 0.985 | 2.25 (0.78-5.12) | 0.135 |
| Female | 0.97 (0.54–2.90) | 0.972 | - | - |
| Renal insufficiency | 2.46 (1.05–4.12) | 0.031 | - | - |
| Baseline PTH (per doubling) | 3.07 (1.92–4.09) | <0.001 | 2.02 (1.39–4.09) | 0.016 |
| Serum calcium (mmol/L) | 7.03 (3.03–22.71) | <0.001 | 1.87 (0.58–5.23) | 0.367 |
| Serum phosphorus (mmol/L) | 0.013 (0.002–0.14) | <0.001 | - | - |
| 25(OH)D3 (ng/mL) | 1.11 (1.06–1.17) | 0.003 | 1.08 (1.02–1.16) | 0.044 |
| Albumin (g/L) | 0.91 (0.86–0.97) | 0.037 | - | - |
| Alkaline phosphatase (U/L) | 1.01 (1.00–1.01) | <0.001 | 1.00 (1.00–1.01) | 0.372 |
| LDL cholesterol (mmol/L) | 0.66 (0.48–0.90) | 0.069 | - | - |
| Maximum gland diameter (cm) | 1.86 (1.43–2.45) | 0.002 | 1.09 (0.74–1.47) | 0.668 |

Abbreviations: MWA, microwave ablation; PTX, parathyroidectomy; PTH, parathyroid hormone; OR, odds ratio; CI, confidence interval;
